# Editorial: Molecular chaperones and polyamines in disease

**DOI:** 10.3389/fmolb.2025.1658176

**Published:** 2025-07-09

**Authors:** Stanley Makumire, Graham Chakafana, Xolani H. Makhoba

**Affiliations:** ^1^ Structural Biology Research Unit, Faculty of Biochemistry and Molecular Medicine, University of Oulu, Oulu, Finland; ^2^ Department of Chemistry and Biochemistry, Hampton University, Hampton, VA, United States; ^3^ Department of Life and Consumer Sciences, College of Agriculture and Environmental Sciences, University of South Africa (UNISA), Roodepoort, South Africa

**Keywords:** molecular chaperones, polyamines, proteostasis, biomarkers, therapeutic targets

Molecular chaperones and polyamines are essential for cellular proteostasis and function. Chaperones facilitate proper protein folding, assembly, translocation, and degradation, while polyamines modulate nucleic acid structure, translation, and cellular stress responses. The functional interplay between these systems is crucial for cellular homeostasis and has been implicated in the progression of various diseases, including cancer, neurological disorders, and metabolic conditions. Our call focused on understanding the mechanisms behind their influence on disease pathogenesis and their potential as therapeutic targets or biomarkers, to advance the development of novel therapeutics and deepen insights into cellular regulation.

In this regard, a study by Zayed et al. evaluated the therapeutic potential of a known Hsp70 activator, SW02, for treating prion and neurodegenerative diseases. Using both *in vitro* and *in vivo* methods, their findings suggest that SW02 shows promise as a neuroprotective agent, particularly through its effects on protein homeostasis and prion burden. On the other hand, Smith et al. investigated the role of different human Hsp90 isoforms in neuroinflammation, identifying Hsp90β as a key driver of neuroinflammation, suggesting it as a more selective and safer drug target for developing anti-inflammatory or pain management therapies, potentially avoiding side effects seen with non-selective Hsp90 inhibitors.

Recently, studies have focused on the critical role of polyamine metabolism in disease. In our call, studies concentrate on polyamine metabolism as a central driver of hepatocellular carcinoma (HCC) development, immune suppression, and resistance to therapy, focusing on its impact on prognosis, tumor behavior, immune regulation, and response to immunotherapy. Wu et al. review the important role of polyamines (putrescine, spermidine, and spermine) in cancer development and immune regulation. They discuss how polyamine metabolism, which differs between normal and cancerous tissues, contributes to tumor growth, metastasis, and immune evasion. Polyamines affect both tumor cells and immune cells in the tumor microenvironment, influencing responses to immunotherapy. The review also examines how polyamine-related changes in metabolism and epigenetics can lead to resistance against immune checkpoint inhibitors. It also explores therapeutic strategies targeting polyamine pathways, particularly in combination with immunotherapy, as a promising approach for cancer treatment.

Interestingly, using 101 machine learning algorithms to identify 9 key genes, Yu et al. developed a prognostic risk signature for hepatocellular carcinoma (HCC) based on polyamine metabolism. The signature is highly predictive, revealing distinct differences in clinical traits, biological activity, mutation patterns, and immune cell infiltration between high- and low-risk groups. Immune analyses (TIDE and IPS) indicated differing responses to immunotherapy between the two groups. RT-qPCR validation showed that the 9 genes were highly expressed in normal cells but downregulated in tumor cells. Overall, their findings provide a potential tool for guiding personalized treatment in HCC patients.

Furthermore, Pan et al. explored the role of polyamine metabolism in hepatocellular carcinoma (HCC), identifying two patient clusters with different survival outcomes based on polyamine-related genes. They also developed a prognostic signature called PAscore, which effectively predicted poor prognosis, low immune cell infiltration, and reduced sensitivity to immunotherapy. Malignant HCC cells showed heterogeneity in polyamine metabolism, with high-activity cells displaying altered pathway activity and increased interaction with myeloid cells. *In vitro*, they identified the FIRRE gene as a key driver of tumor growth and proliferation. Overall, the study underscores PAscore as a valuable tool for predicting outcomes and immunotherapy responses in HCC, while revealing metabolic diversity within tumor cells that shapes the tumor microenvironment.

Collectively, the contributions to this call highlight the multifaceted roles of molecular chaperones and polyamines in regulating cellular homeostasis and their emerging significance in disease pathogenesis. As our understanding of these systems deepens, the intersection of molecular chaperone biology and polyamine signaling offers novel opportunities for biomarker development and the design of targeted interventions in cancer and neurological disorders. We anticipate that continued exploration in this field will pave the way for innovative therapeutic strategies that leverage the critical regulatory roles of chaperones and polyamines in human health and disease.

